# Novel Insulin Sensitizer Modulates Nutrient Sensing Pathways and Maintains β-Cell Phenotype in Human Islets

**DOI:** 10.1371/journal.pone.0062012

**Published:** 2013-05-01

**Authors:** Nidhi Rohatgi, Haytham Aly, Connie A. Marshall, William G. McDonald, Rolf F. Kletzien, Jerry R. Colca, Michael L. McDaniel

**Affiliations:** 1 Department of Pathology and Immunology, Washington University in St. Louis, St. Louis, Missouri, United States of America; 2 Metabolic Solutions Development Company, Kalamazoo, Michigan, United States of America; Omaha Veterans Affairs Medical Center, United States of America

## Abstract

Major bottlenecks in the expansion of human β-cell mass are limited proliferation, loss of β-cell phenotype, and increased apoptosis. In our previous studies, activation of Wnt and mTOR signaling significantly enhanced human β-cell proliferation. However, isolated human islets displayed insulin signaling pathway resistance, due in part to chronic activation of mTOR/S6K1 signaling that results in negative feedback of the insulin signaling pathway and a loss of Akt phosphorylation and insulin content. We evaluated the effects of a new generation insulin sensitizer, MSDC-0160, on restoring insulin/IGF-1 sensitivity and insulin content in human β-cells. This novel TZD has low affinity for binding and activation of PPARγ and has insulin-sensitizing effects in mouse models of diabetes and ability to lower glucose in Phase 2 clinical trials. MSDC-0160 treatment of human islets increased AMPK activity and reduced mTOR activity. This was associated with the restoration of IGF-1-induced phosphorylation of Akt, GSK-3, and increased protein expression of Pdx1. Furthermore, MSDC-0160 in combination with IGF-1 and 8 mM glucose increased β-cell specific gene expression of *insulin, pdx1, nkx6.1,* and *nkx2.2,* and maintained insulin content without altering glucose-stimulated insulin secretion. Human islets were unable to simultaneously promote DNA synthesis and maintain the β-cell phenotype. Lithium-induced GSK-3 inhibition that promotes DNA synthesis blocked the ability of MSDC-0160 to maintain the β-cell phenotype. Conversely, MSDC-0160 prevented an increase in DNA synthesis by blocking β-catenin nuclear translocation. Due to the counteracting pathways involved in these processes, we employed a sequential ex vivo strategy to first induce human islet DNA synthesis, followed by MSDC-0160 to promote the β-cell phenotype and insulin content. This new generation PPARγ sparing insulin sensitizer may provide an initial tool for relieving inherent human islet insulin signaling pathway resistance that is necessary to preserve the β-cell phenotype during β-cell expansion for the treatment of diabetes.

## Introduction

Types 1 and 2 diabetes are associated with reduced β-cell mass and diminished function that prevents normal glucose homeostasis [1], [2]. Major bottlenecks in the expansion of human β-cell mass are limited levels of proliferation, the loss of β-cell phenotype and increased apoptosis [3]–[5]. Our previous studies demonstrated that nutrient activation of Mammalian Target of Rapamycin (mTOR) enhanced DNA synthesis, cell cycle progression and β-cell proliferation in isolated rodent islets. In contrast, isolated human islets displayed insulin signaling pathway resistance mediated, in part, by chronic over activation of mTOR/S6K1 signaling that resulted in the loss of Akt phosphorylation in response to nutrients and growth factors [6], [7]. This inhibition of the insulin signaling pathway prevented the engagement of the Wnt/GSK-3/β-catenin pathway essential for β-cell proliferation [8]. We circumvented the insulin signaling pathway resistance in human islets by pharmacologic inhibition of GSK-3 that increased Wnt signaling, significantly increasing β-cell proliferation. However, the problem of insulin signaling pathway resistance due to chronic mTOR activation was still present, resulting in a loss of insulin content [7]. It has been suggested that there may be a switch mechanism between insulin secretory granule production and proliferation [9]. Thus, the decrease in insulin stores may be reversible under appropriate recovery conditions [10]–[13].

Although none of the islet donors were diagnosed with type 2 diabetes, nearly all of the cadaver-derived human islets that we receive display insulin signaling pathway resistance as determined by decreased response to exogenous insulin or IGF-1. The reasons for this resistance are unclear but may be the result of in vitro culture or isolation and shipping conditions that result in hypoxia-induced stress and chronic activation of mTOR that can adversely affect cell survival [7], [14]. Thus, it was necessary to reduce chronic mTOR activation that was associated with insulin signaling pathway resistance in human islets. Rapamycin, a highly selective allosteric and potent inhibitor of mTOR, reduced negative feedback and restored Akt signaling [7], but is not a viable candidate for the physiological modulation of mTOR and preservation of insulin content [15]. Rapamycin at low nM concentrations inhibited β-cell proliferation and mTOR-mediated nuclear translocation of the transcription factor, β-catenin, necessary for proliferation in human β-cells, although β-cell function did not change [7], [8]. Importantly, inhibition of mTOR by rapamycin is also not readily reversible, impairs glucose homeostasis, and inhibits both mTORC1 and mTORC2 without adequate specificity [15], [16]. To address the loss of β-cell phenotype, in particular, insulin content and to restore the insulin signaling pathway, we needed to identify an alternative method to downregulate mTOR as opposed to the use of allosteric or catalytic inhibitors of mTOR.

The thiazolidinediones (TZDs), rosiglitazone and pioglitazone, currently used clinically to improve insulin resistance in type 2 diabetes, are proposed to exert their effects through PPARγ receptor-mediated gene transcription in adipose tissue [17], and are also thought to preserve β-cell mass and function [18]. Despite anti-diabetic properties, rosiglitazone and pioglitazone cause use-limiting side effects related to PPARγ activation including increased adiposity, edema, an increased rate of fractures of the small bones of extremities, and in addition cardiovascular issues with rosiglitazone [19]–[21].

Recently, it has been recognized that TZDs, in addition to activating the nuclear receptor PPARγ, also exert physiological effects by modifying mitochondrial metabolism and activating AMPK [22]–[27]. Our previous findings indicated that appropriate regulation of the mitochondrial TCA cycle is not only important for insulin secretion but may significantly impact the growth and proliferation of β-cells through mTOR-dependent signaling pathways [16]. Thus, the use of TZDs may be a feasible approach to regulate mitochondria and mTOR. In agreement with this hypothesis, Gleason et al. [26] also identified a role for mitochondrial metabolism and decreased AMPK in the regulation of nutrient-stimulated mTOR activation.

In this study, we evaluated the ability of a new generation TZD specifically designed for diminished PPARγ binding [27], [28], to modulate mTOR, restore insulin content, and maintain a differentiated state in human islet β-cells in vitro. We developed a sequential strategy consisting of Wnt pathway activation to induce DNA synthesis followed by MSDC-0160 treatment to promote insulin content and maintain a β-cell phenotype. This approach may provide a balance between the pathways that regulate growth and phenotype in the expansion of human β-cell mass ex vivo. In agreement with the findings of Chen et al. that a PPARγ sparing TZD improves insulin pathway sensitivity in multiple tissues including striated muscle, adipose tissue and liver [27], we now demonstrate that this also occurs in human islet cells.

## Materials and Methods

### Materials

MSDC-0160 (also known as PNU-91325 [28]), was from Metabolic Solutions Development Company (Kalamazoo, MI, USA); lithium chloride (LiCl) was from Sigma (St. Louis, MO, USA), IGF-1, forskolin, RIA kit from EMD Millipore (Billerica, MA, USA); rapamycin and LY294002 from Enzo Life Sciences (Farmingdale, NJ, USA), ^3^H-thymidine (Perkin-Elmer, Waltham, MA, USA); Nupage 10% or 4–12% Bis-Tris gels (Life Technologies, Carlsbad, CA, USA); Immun-Star WesternC chemilumiscence kit (Bio-Rad, Hercules, CA, USA).

### Human Islets

Human islets were obtained from the Integrated Islet Distribution Program (IIDP) sponsored by the NIDDK and the JDRFI and also the JDRFI Sponsored Islets for Research Program at Washington University (JDRF-31-2008-382). The IIDP uses only cadaver donors that have consented to research. The Washington University Medical Center (WUMC) Human Studies Committee (HSC) IRB approved all studies involving the use of isolated cadaver-derived human islets (Approval number: 93-0068). The IRB exempted the study from HIPAA compliance based on regulatory definition of human subject. Review date: 7/8/2004; review committee: 08 MRCR. Islets were cleaned and cultured overnight at 37°C in “complete” CMRL-1066 medium (cCMRL, containing 5.6 mM glucose, 10% FBS, 100 U/ml penicillin, 100 µg/ml streptomycin, 2 mM L-glutamine). Islets were handpicked for experiments. Human islet donor data: 55 donors; 25 males, 26 females, 4 not documented; age, mean 44.10 yrs (range 19–63); BMI, mean 26.39 (range 18.8–40.9); purity, mean 94.13% (range 60–99%); and viability, mean 81.5% (range 50%–99%). None of the donors were diagnosed with diabetes.

### Rat Islet Isolation

Rat islets were obtained from adult male Sprague-Dawley rats (250 g; Harlan Sprague-Dawley) as previously described [7]. Studies were approved by the Washington University Animal Studies Committee.

### Real Time Polymerase Chain Reaction

Islets (75 per dish) were cultured in Petri dishes with 1 ml cCMRL and treatment conditions as indicated in the figure legends. Total RNA was extracted using RNeasy Mini Kit (Qiagen, Valencia, CA, USA) and ∼500 ng was used for cDNA synthesis using High Capacity cDNA Reverse Transcription Kit (Life Technologies, Carlsbad, CA, USA). qRT-PCR was performed in triplicate (Taqman gene expression master mix and fluorigenic probes, StepOnePlus Real-Time PCR System Applied Biosystems, part of Life Technologies, Carlsbad, CA, USA). Relative quantitative analysis was performed according to the comparative CT method using the arithmetic formula 2^(ΔΔct)^. cDNA levels were normalized to human β-actin cDNA.

### Immunohistochemistry

Human islets 50–60 were treated for 24 hours or 4 days. Islets were then dispersed as described previously [7]. Cells were fixed in 4% paraformaldehyde and permeabilized using 0.5% Triton X-100 for 15 min. Cells were blocked using blocking buffer (5% normal donkey serum/0.1% Triton X-100/1X PBS) for 1 h at room temperature. Indirect immunofluorescence was performed using primary antibodies, guinea pig anti-insulin (Dako, Carpinteria, CA, USA), rabbit anti-Pdx1 (Cell Signaling, Danvers, MA, USA) diluted in blocking buffer and secondary antibodies, Alexa 488 donkey anti-guinea pig (Jackson Immunoresearch, West Grove, PA, USA) and Alexa 568 donkey anti-rabbit (Life Technologies, Carlsbad, CA, USA). Cells were counterstained and mounted using ProLong antifade gold reagent with 4′,6-diamindino-2-phenylindole (DAPI, Life Technologies, Carlsbad, CA, USA).

For Pdx1 and β-catenin localization in whole islets, paraffin embedded islet sections underwent antigen retrieval by microwaving sections in citrate buffer (10 mM) for 15 min. Sections were blocked using 5% normal donkey serum/0.1% Triton X-100/1X PBS and labeled with primary antibodies, rabbit anti-Pdx1 or rabbit anti-β-catenin (Cell Signaling, Danvers, MA, USA) and guinea pig anti-insulin (Dako, Carpinteria, CA, USA) diluted in blocking buffer. Secondary antibodies used were same as mentioned above. Images were acquired with a Nikon TE300 microscope equipped with Photometric Coolsnap ES2 camera and Metamorph v 7.6.5.0 software.

### For Mpc2 (BRP44) and Mpc1 (BRP44-Like) Protein

Human islets were dispersed as mentioned above and indirect immunofluorescence was performed using primary antibodies, guinea pig anti-insulin (Dako, Carpinteria, CA, USA), rabbit anti-BRP44 (also known as Mpc2; MSDC, Kalamazoo, MI, USA), rabbit anti-BRP44-Like (also known as Mpc1; Abcam, Cambridge, MA, USA) and secondary antibodies, Alexa 488 donkey anti-guinea pig (Jackson Immunoresearch, West Grove, PA, USA) and Alexa 568 donkey anti-rabbit (Life Technologies, Carlsbad, CA, USA). Cells were counterstained and mounted using ProLong antifade gold reagent with 4′,6-diamindino-2-phenylindole (DAPI, Life Technologies, Carlsbad, CA, USA). For cells stained for Mpc2/Mpc1 protein with Mitotracker, islets were treated for 30 min with CMRL containing 500 nM CMXRos Mitotracker red probe according to the manufacturer’s protocol. Images were acquired with a Nikon TE300 microscope equipped with Photometric Coolsnap ES2 camera and Metamorph v 7.6.5.0 software.

### Mitochondrial P2 Fractions

Human islets (500–700) were homogenized in 10× volume of cold fractionation buffer (50 mM Tris, 250 mM sucrose, pH 8.0) with 15 strokes of Dounce homogenizer. Islets were centrifuged 1000×g for 5 min at 4°C. Pellet (P1) fractions were re-homogenized in same volume as original homogenate and centrifuged at 1000×g for 5 minutes. Post nuclear supernatants (S1s) were combined and centrifuged at 20,000×g for 20 minutes at 4°C. Pellet (P2) was resuspended in 50 mM Tris pH 8.0.

### Binding of MSDC-0160 to its Mitochondrial Target Protein (mTOT)

Human islets (∼1000–1500/sample) were homogenized, and 10 µg of protein (mitochondrial P2 fraction) was incubated with a ^125^I-labeled probe (MSDC-1101) that binds to Mpc2 with or without the concomitant addition of 20 µM MSDC-0160 as previously described [23], [27].

### Western Blotting

Islets (150) were incubated in 1 ml cCMRL and treated as indicated in the figure legends. Islets were sonicated in lysis buffer containing phosphatase and protease inhibitors (Active Motif, Carlsbad, CA, USA). Cell debris was removed by centrifuging samples for 10 min at 4°C. Extracts (10–30 µg) were resolved using Nupage 10% or 4–12% Bis-Tris gel and transferred to nitrocellulose membranes. Membranes were probed using antibodies against: phospho-AMPKα (Thr172), phospho-Akt (Ser473), phospho-Akt (Thr308), phospho-GSK-3α/β, phospho-mTOR (Ser2448), Pdx1, phospho-S6 (Ser235/236) and total Akt, GSK-3β and S6 (All antibodies raised in rabbit, Cell Signaling, Danvers, MA, USA), rabbit phospho-ACC (Ser79) (EMD Millipore; Billerica, MA, USA), rabbit anti-BRP44, also known as Mpc2 (MSDC, Kalamazoo, MI, USA), and β-actin (Sigma, St. Louis, MO, USA) according to supplier’s protocols. Horseradish peroxidase-conjugated donkey anti-rabbit or anti-mouse IgG (Jackson Immunoresearch, West Grove, PA, USA) were used as secondary antibodies with Immun-Star WesternC chemiluminescence for detection. A ChemiDoc XRS^+^ with Image lab software (BioRad, Hercules, CA, USA) was used to visualize and quantify the protein expression.

### ^3^H-Thymidine Incorporation

Islets (100) were cultured in Petri dishes for 4 days in 1 ml of cCMRL with treatment conditions as indicated in the figure legends at 37°C. During the final 24 h of the 96 h incubation, 10 µCi of ^3^H-thymidine was added, and ^3^H-thymidine incorporation determined [16].

### Insulin Secretion

Islets were cultured in cCMRL with 8 mM glucose ± MSDC-0160 at 37°C. After 4 days, MSDC-0160 was removed and islets incubated for 90 min in cCMRL with 5 mM glucose. Insulin secretion was measured by static incubation for 30 min with cCMRL containing 5 or 16.5 mM glucose ± forskolin. Triplicate samples of 10 islets for each treatment were used. Media were collected and insulin determined by Human Insulin Specific RIA.

### Insulin Content

Islets were received and immediately cleaned in cCMRL, time-0 samples counted and processed for insulin content. Remaining islets were counted into dishes and cultured for 4 days in 1 ml cCMRL with treatment conditions. At the end of the incubation, 20 islets in duplicate from each treatment were handpicked and washed with PBS +0.05% BSA. Islets were sonicated in the same buffer, and total insulin content determined by RIA.

### Expression of Data and Statistics

Data are presented as mean ± SEM. Statistically significant differences were assessed with unpaired t-tests, one-way or two-way ANOVA followed by Newman-Keuls Multiple Comparison Test. Significant differences indicated by *(p<0.05), **(p<0.01) and ***(p<0.001).

## Results

### Human islets do not Increase DNA Synthesis in Response to Nutrients or Growth Factors in vitro

**Figure 1:**
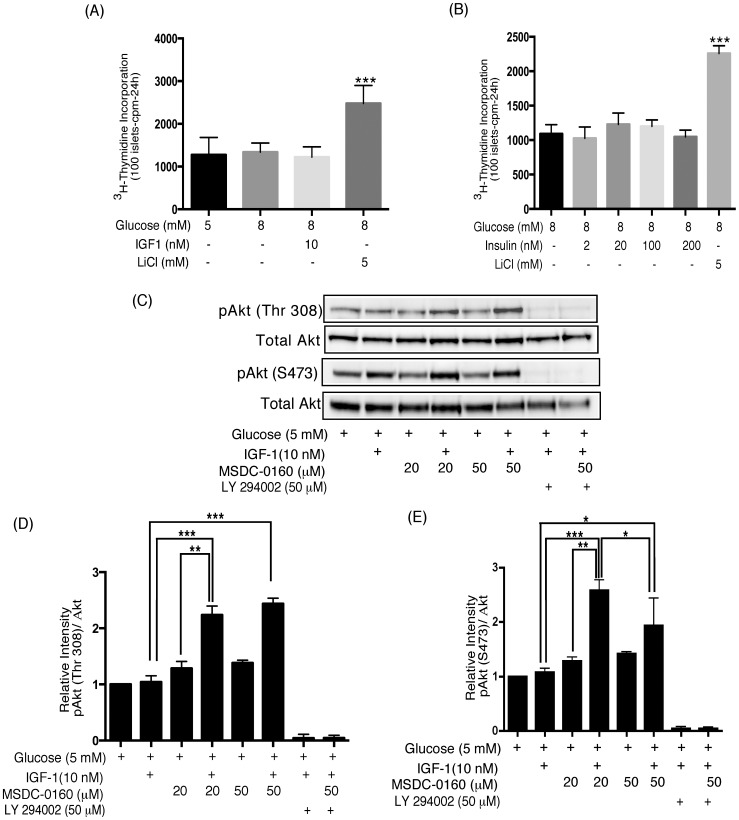
Human islets display insulin signaling pathway resistance. (**A, B**) Human islets do not increase DNA synthesis in response to nutrients or insulin/IGF-1 in vitro. Human islets (100) were cultured for 4 days in cCMRL, 8 mM glucose+MSDC-0160, insulin (A) or IGF-1 (B) and LiCl as indicated. ^3^H-thymidine was added to each dish 24 h before the end of the 4-day period. Data are the means±SEM from n = 3, with duplicate samples in the experiment. Data analyzed using one-way ANOVA followed by Newman-Keuls Post-hoc test. (**C–E**) PPARγ sparing TZD, MSDC-0160, restores the insulin-signaling pathway. Human islets were cultured for 90 min in cCMRL, 5 mM glucose with or without the concentrations of MSDC-0160, IGF-1, LY 294002 as indicated. Cell extracts were prepared and samples were processed for Western blotting and quantitated by densitometry. Following detection of the phosphorylated proteins, the Western blots were reprobed with antibodies against their respective endogenous control (Total Akt). In each figure, the upper panel (**Fig. C**) shows representative Western blots and the lower panels (**Fig. D&E**) shows mean values after quantitation of the data. Data analyzed using one-way ANOVA followed by Newman-Keuls test. Data are means±SEM of n = 3 (D) or n = 4 (E) experiments.

Figures 1A&B demonstrate that isolated human islets fail to increase DNA synthesis in response to an increased glucose concentration, exogenous insulin or IGF-1. LiCl that directly inhibits GSK-3 and engages the Wnt signaling pathway, significantly increased DNA synthesis in the same set of human islets. We have previously shown that rodent islets significantly increase DNA synthesis in response to an increase in glucose or exogenous insulin [7] suggesting that human islets may display resistance in the insulin/IGF-1 signaling pathway.

### PPARγ Sparing TZD, MSDC-0160, Reduces Resistance in the Insulin/IGF-1 Signaling Pathway and Restores IGF-1-induced Akt Phosphorylation

To determine if the insulin signaling pathway had been restored by drug treatment, we evaluated the effects of acute (90 min) MSDC-0160 treatment on IGF-1 mediated phosphorylation of Akt. In Fig. 1C–E, IGF-1 alone failed to enhance Akt at two distinct phosphorylation sites, Thr308 and Ser473 (columns 1 vs. 2). However, co-incubation of islets with MSDC-0160+ IGF-1 to activate the insulin-signaling pathway, resulted in a significant increase in Akt (Fig. 1C–E columns 2 vs. 4 and 6) and GSK-3β (Ser9) phosphorylation (not shown). Pretreatment with LY294002, an inhibitor of PI-3 K, completely blocked MSDC-0160 and IGF-1 induced Akt phosphorylation (Fig. 1C columns 7 and 8), indicating that the insulin signaling pathway is a major source of Akt (Thr308/Ser473) activation at basal glucose.

### MSDC-0160 in Combination with IGF-1 Treatment Prevents the Loss of Insulin Content

**Figure 2:**
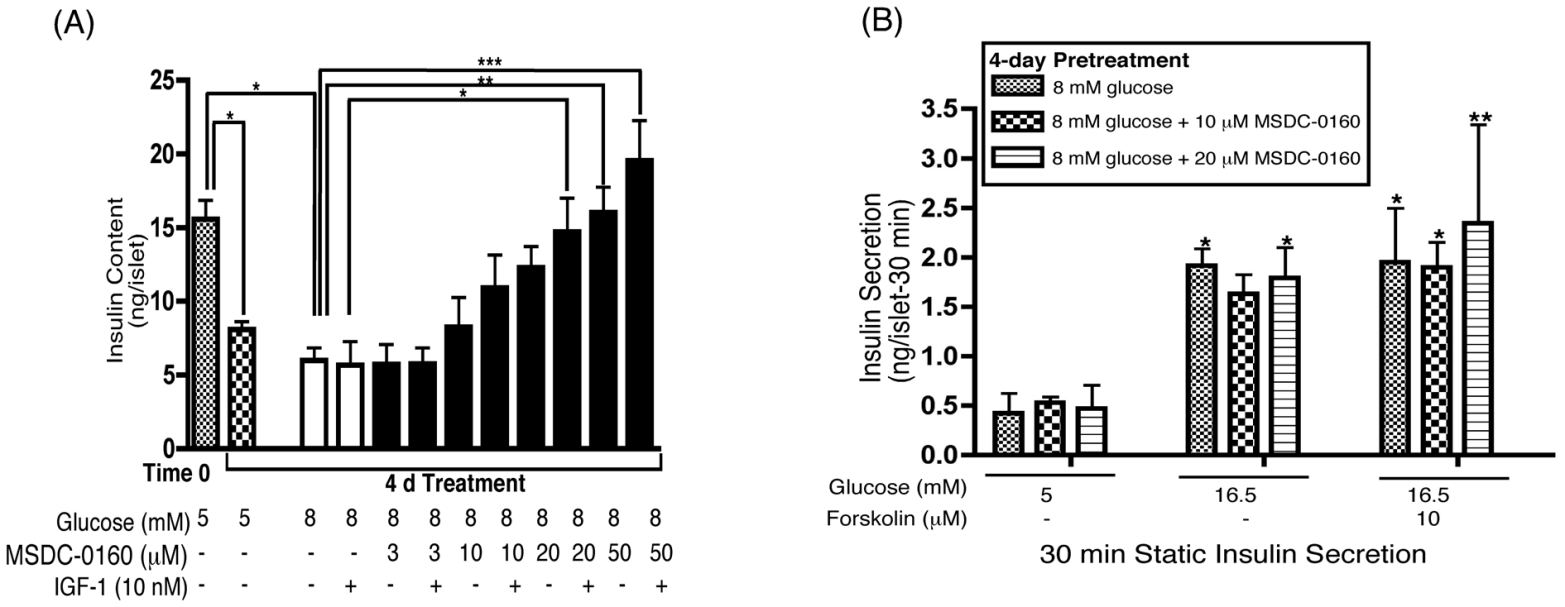
Treatment of human islets with MSDC-0160± IGF-1 prevents the loss of insulin content and maintains insulin secretion. Islets were cultured in cCMRL, 5 or 8 mM glucose with or without the concentrations of MSDC-0160± IGF-1 for 4-days as indicated. (**A**) Islet insulin content was measured from 20 islets in duplicate at the start of the incubation period (time-0) and after 4-days of incubation under the conditions shown. Islets were sonicated in PBS with 0.05% BSA and kept at −20°C until assayed as described. (**B**) Islets were cultured in cCMRL with 8 mM glucose ± MSDC-0160. After 4 days, MSDC-0160 was removed and islets incubated for 90 min in cCMRL with 5 mM glucose. Insulin secretion was measured by a static incubation for 30 min with cCMRL containing 5 or 16.5 mM glucose ± forskolin. Triplicates of 10 islets were assayed. Insulin was measured by radioimmunoassay. Data are means±SEM from n = 3 (A) and n = 2 (B) with duplicate (A) or triplicate (B) samples in each experiment. Data was analyzed using one-way ANOVA followed by Newman-Keuls Post-hoc test (A) and two-way ANOVA followed by Bonferroni Post-hoc test (B).

As shown in Fig. 2A, insulin content in human islets decreased ∼50% during a 4 day incubation at 5 or 8 mM glucose in comparison to insulin content at time-0. IGF-1 alone failed to preserve insulin content, whereas IGF-1 in combination with 20 or 50 µM MSDC-0160 was statistically significant. In addition, MSDC-0160 at 50 µM alone was also significant. In Fig. 2B, human islets were pretreated with 8 mM glucose ± MSDC-0160 (10–20 µM) for 4 days, and then the pretreatment media containing MSDC-0160 was removed. Following a 90 min incubation period at 5 mM glucose, an acute static insulin secretion assay was performed. These findings suggest that pretreatment with MSDC-0160 did not alter subsequent insulin secretion and the islets remained physiologically functional in response to 16.5 mM glucose ± forskolin compared to 5 mM glucose.

### Treatment of Human Islets with MSDC-0160 Activates AMPK and Downregulates mTOR

**Figure 3:**
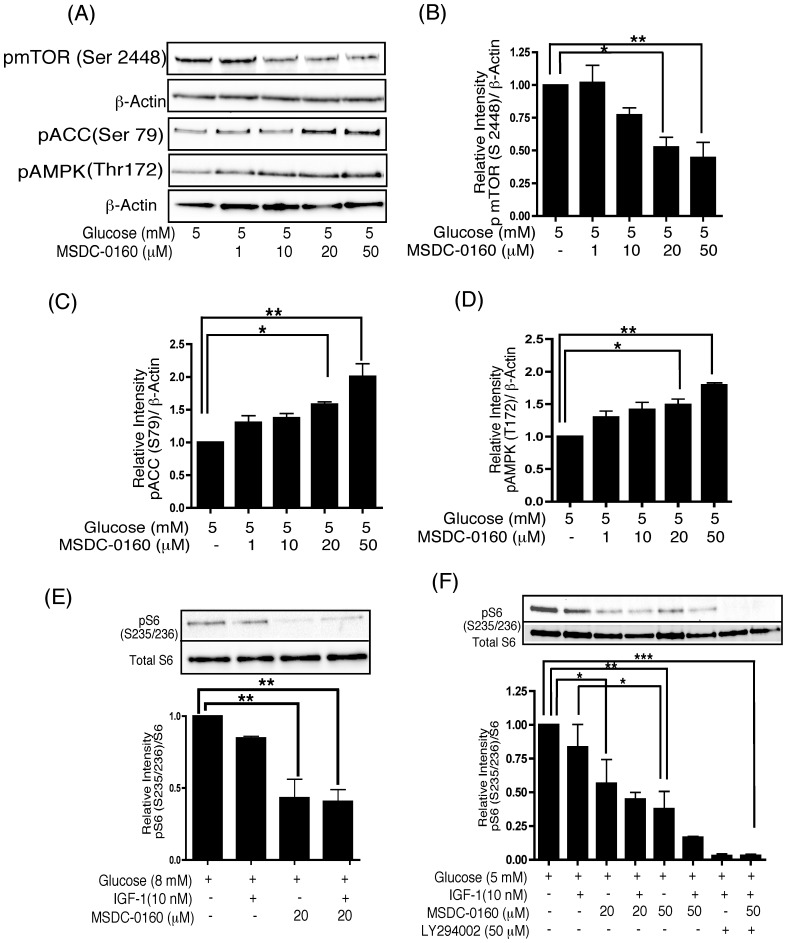
(A–D) Treatment of human islets with MSDC-0160 activates AMPK and downregulates mTOR. Islets were cultured for 24 h in cCMRL, 5 mM glucose±MSDC-0160 as indicated. Cell extracts were prepared and samples processed for Western blotting and quantitated by densitometry. β-actin was used as a protein loading control. (**A**) Representative Western blot for activated mTOR, AMPK and downstream target of AMPK, ACC. (**B–D**) Densitometry of Western blots shown in (A) for phosphorylated mTOR, pACC and pAMPK, respectively. Data are means±SEM of n = 2 experiments. Data analyzed using one-way ANOVA followed by Newman-Keuls test. (**E–F**) MSDC-0160 downregulates mTOR target, pS6 to relieve mTOR-mediated negative feedback. Human islets were cultured for 4 days (E) or 90 min (F) in cCMRL, 5 or 8 mM glucose with or without the concentrations of MSDC-0160, IGF-1, LY294002 as indicated. Cell extracts were prepared, samples processed for Western blotting and quantitated by densitometry. Following detection of the phosphorylated proteins, the Western blots were reprobed with total S6 antibodies, used as endogenous control. In each figure, the upper panel shows a representative Western blot and the lower panel shows mean values after quantitation of the data. Data analyzed using one-way ANOVA followed by Newman-Keuls test. Data are means±SEM of n = 3 (E) or n = 4 (F) experiments.

Human islets were cultured with 5 mM glucose and MSDC-0160 (1–50 µM) for 24 h. Phosphorylation of mTOR was significantly decreased at 20 and 50 µM MSDC-0160 (Fig. 3A&B). This inhibition inversely correlated with increased phosphorylation of AMPK and its downstream target acetyl CoA carboxylase (ACC) (Fig. 3A, C, D). The effects of MSDC-0160 at 5 mM glucose were similar at 8 mM glucose (data not shown). Consistent with the reduction in the amount of phosphorylated mTOR, treatment with MSDC-0160 also produced a decrease in the phosphorylation of S6, a downstream indicator of the mTOR pathway. This was evident after 4 days of culture under these conditions (Fig. 3E), but occurred as early as 90 minutes into the treatment (Fig. 3F). Taken together, these data suggest that MSDC-0160 restores the insulin signaling pathway at least in part by modulating mTOR activity.

### MSDC-0160 Treatment Maintains Human β-cell Phenotype

**Figure 4:**
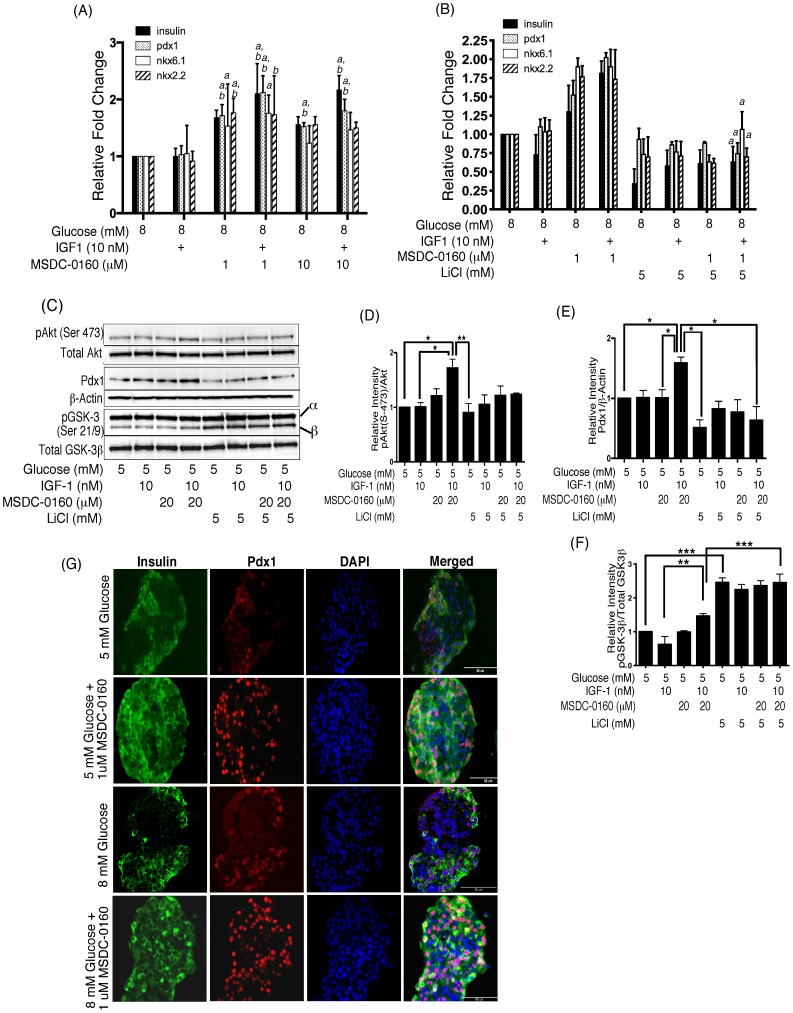
MSDC-0160+ IGF-1 maintains human β-cell phenotype. (**A–B**) MSDC-0160 treatment increases expression of β-cell specific genes. Human islets were cultured for 24 h in cCMRL, 8 mM glucose with or without the concentrations of MSDC-0160, IGF-1 and LiCl as indicated. Islets were harvested for RNA isolation, cDNA synthesis and real-time PCR analysis. Taqman probes with primers specific for *insulin, pdx1, nkx6.1* and *nkx2.2* genes were used. Target genes were quantified by relative quantification using the comparative Ct method. Data are means±SEM of n = 3–4 experiments. In (A), symbols a = p≤0.05 with respect to 8 mM glucose; b = p≤0.05 with respect to 8 mM glucose+IGF1. In (B), a = p≤0.05 with respect to 8 mM glucose+MSDC-0160+ IGF-1. (**C–F**) Activation of Wnt pathway by LiCl blocks the effects of MSDC-0160 on the insulin-signaling pathway. Western Blots for phosphorylated Akt, Pdx1 and pGSK-3β were normalized to total proteins (Akt, GSK-3β) or β-actin. Left panels show representative Western blots and right panels show mean values after quantitation of the densitometry. Data are means±SEM from n = 4 experiments. Data was analyzed using one-way ANOVA followed by Newman-Keuls test. Symbols * represent p<0.05, ** p<0.01, *** p<0.001. Incubation of islets for 24 h with MSDC-0160+ IGF-1 resulted in significant increase in phosphorylation of Akt, and Pdx1, in comparison to IGF-1 alone (lanes 2 vs. 4). In contrast, LiCl alone had no significant impact on the insulin-signaling pathway (lane 5), but blocked the ability of MSDC-0160± IGF-1 to increase phosphorylation of Akt (Figs. 2A&B, lanes 5–8) and Pdx1 protein expression. (**G**) Immunohistochemical staining of insulin and Pdx1 in human islets. Human islets were cultured for 24 h in cCMRL containing 5 or 8 mM glucose±MSDC-0160 as indicated on the left of the figure. Islets were embedded in paraffin and 5 µm thick paraffin sections were processed for IHC staining as described in methods. Images were acquired at 40X magnification and are representative of n = 3 experiments. Scale bar = 50 µm. Insulin = green, Pdx1 = Red, DAPI = blue.

Gene expression of markers of the β-cell phenotype including *insulin*, *pdx1, nkx6.1*, and *nkx2.2*, did not increase in response to 8 mM glucose+IGF1 compared to 8 mM glucose alone (Fig. 4A&B). MSDC-0160 (1 and 10 µM) ± IGF1 significantly increased the gene expression above 8 mM glucose ± IGF-1 (Fig. 4A). Levels of NeuroD1 did not change (data not shown). Importantly, inhibition of GSK-3 by LiCl by augmenting its phosphorylation, blocks these positive effects of MSDC-0160 on gene expression under all conditions (Fig. 4B, columns 5–8). Neither IGF-1 nor MSDC-0160 (1 µM) was able to overcome this decrease in gene expression due to LiCl. Similarly, insulin (200 nM)+MSDC-0160 (50 µM) increased gene expression of *insulin* and *pdx1*, that was blocked by the addition of LiCl (Fig. S1). These experiments were performed with 5 and 8 mM glucose with the same results (Fig. S1). In Fig. 4, islets cultured with MSDC-0160+ IGF-1 for 24 h increased pAkt (Ser473) (Fig. 4C&D) and expression of Pdx1 (Fig. 4C, E). In contrast, inhibition (increased phosphorylation) of GSK-3 by LiCl (confirmed in Fig. 4F) blocked these effects. Thus, MSDC-0160 and IGF-1 significantly increase β-cell specific gene expression in human islets, and inhibition of GSK-3 blocks these positive effects. Immunohistochemical (IHC) analysis of human islets confirmed that MSDC-0160 at concentrations as low as 1 µM for 24 h resulted in an increased expression of β-cell specific Pdx1 and insulin, and the expression of Pdx1 was greater at 8 mM glucose compared to 5 mM glucose (Fig. 4G). Similar results were observed at 4 days (data not shown).

### Effect of MSDC-0160 on the ERK Pathway

Our studies have focused on the phosphorylation of Akt as an indicator of the insulin/IGF-1 signaling pathway. However, additional markers of insulin/IGF-1 signaling may also be affected by MSDC-0160, such as the ERK pathway. Several investigators have described how insulin resistance may be tissue and/or pathway specific. For example, in vascular smooth muscle cells insulin alone increased both pAkt and pERK, whereas the combination of palmitate and insulin resulted in increased pERK but not pAkt [29]. In Min6 cells, glucose induced both Akt and ERK activation [30]. In mouse islets, glucose increased p-ERK1/2 but not p-Akt. In this same study, IGF-1 had no effect on pAkt and did not further increase glucose-induced p-ERK1/2. Insulin (100 nM) did not increase pAkt or ERK1/2. [31]. In endothelial cells, VEGF and insulin were each able to increase activation of ERK1/2 and pioglitazone inhibited both of these effects [32]. In human islets, we found that MSDC-0160± IGF-1 did not significantly increase pERK (Ser 42/44) during a 24 h exposure (Fig. S2). This is in contrast to the positive effects on pAkt in human islets treated with MSDC-0160+ IGF-1 (e.g., see Fig. 1).

### MSDC-0160 Reduces the Expression of Glucose-induced Apoptotic Genes in Human Islets

Since MSDC-0160 downregulated mTOR activity and restored Akt signaling, we assessed its role in reducing glucose-mediated apoptosis by analyzing the expression of genes in the bcl family. The bcl family includes both pro-apoptotic (*bax*) and anti-apoptotic (*bcl2, bclxL*) genes and exerts a pivotal role in regulating apoptosis. A 4-day exposure with elevated glucose (16.5 and 33.3 mM) triggered the apoptotic pathway in human islets as evidenced by the 3–6-fold increase in *bax* expression (Fig. S3A). These conditions that significantly increased *bax* gene expression tended to result in a concomitant reduction of *bcl2* gene expression. Consistent with this gene expression analysis, 16.5 and 33.3 mM glucose significantly increased protein levels of cleaved caspase-3, compared to 5 mM glucose, an event that immediately precedes apoptosis (Fig. S3B). Next, we determined if treatment of human islets with MSDC-0160 would prevent glucose-induced increases in apoptotic gene expression and cleaved caspase-3 protein levels (Fig. S3C–G). At 5 mM glucose, MSDC-0160 did not affect gene expression of *bcl2*, *caspase-3* or *bax* (Fig. S3C). However, as shown in Fig. S3D, 8 mM glucose in combination with MSDC-0160 resulted in a significant increase in *bcl2* gene expression above 8 mM glucose alone, a significant decrease in *caspase-3* expression level, but with no alteration in *bax*. These effects were also observed at higher glucose levels (16.5 mM, Fig. S3E and 33.3 mM, Fig. S3F) where the MSDC-0160 suppression of *bax* was also evident. There was an additive effect of MSDC-0160 with IGF-1 to significantly decrease levels of cleaved caspase-3 protein in islets treated with 16.5 mM glucose for 4-days (Fig. S3G). Thus, MSDC-0160 may exhibit pro-survival effects in human islets by decreasing cleaved caspase-3 and regulating the expression of apoptosis-related genes in response to elevated glucose concentrations.

### Human Islets Express mTOT

Several studies suggest that TZDs may have effects on mitochondrial function and/or biogenesis [33] and this led us to the possibility that MSDC-0160 may be acting at the level of the mitochondria. Fig. S4A demonstrates that MSDC-0160 (20 µM) competes for the binding of a ^125^I-labeled photoaffinity crosslinking probe to Mpc2 (also known as BRP44) a component of the mitochondrial target of thiazolidinediones (mTOT) in human islets (marked by the arrows). In addition, immunoblotting crude mitochondrial pellets with BRP44 antibody confirmed the presence of this target in islets. Fig. S4B shows 14 kDa Mpc2 in the P2 mitochondrial fraction of both rat islets and rat liver tissue. To confirm the cellular localization of this target, we performed immunohistochemical (IHC) analysis for both Mpc2 and Mpc1 proteins, two key members of mTOT, in human islets. Consistent with the Western blot data, Fig. S4C (left panels) demonstrate that Mpc2 and Mpc1 (green) and the mitochondrial marker, Mitotracker (red), colocalized (yellow) in the human islet cells, associated with the mitochondrial location in human β-cells. In Fig. S4C (right panels) Mpc2 and Mpc1 (red) are present in both β-cells (green) and other cell types. These findings suggest that both Mpc2 and Mpc1 are in the mitochondria of human β-cells and also non-β-cells and provide a potential site of action for MSDC-0160.

### The Processes of DNA Synthesis and Maintenance of Insulin Content do not Occur Simultaneously

**Figure 5:**
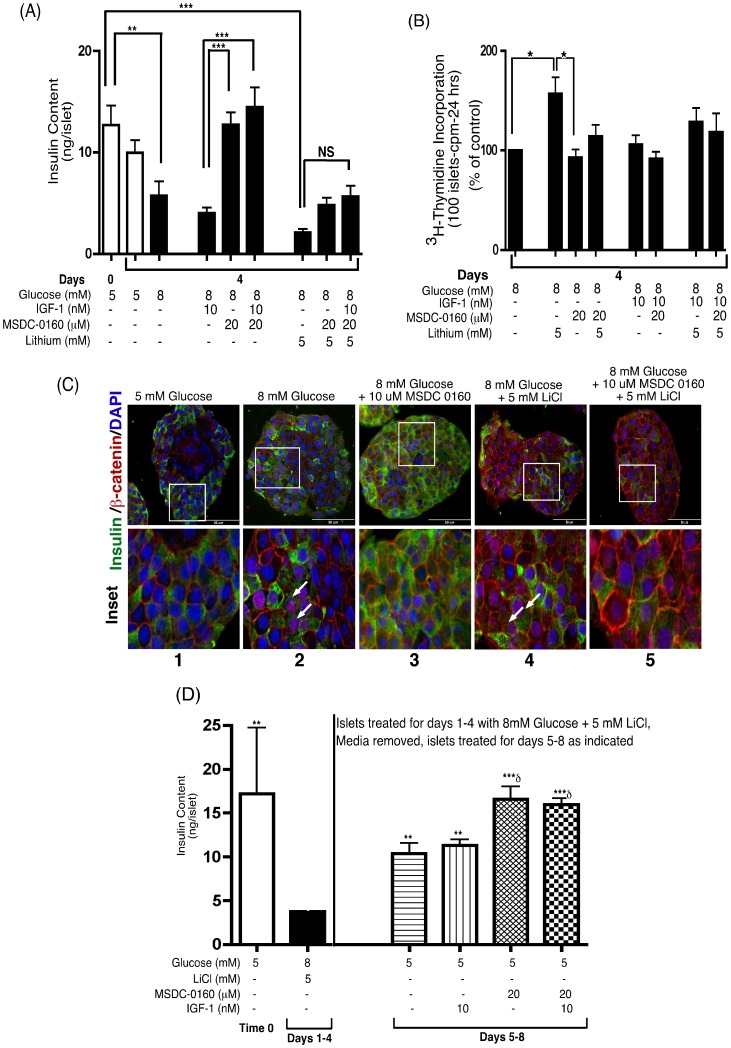
(A, B) The processes of DNA synthesis and maintenance of insulin content do not occur simultaneously. Human islets were cultured in cCMRL, 5 or 8 mM glucose with or without the concentrations of MSDC-0160 and IGF-1 for 4 days as indicated. (**A**) Islet insulin content was measured from 20 islets in duplicate at the start of the incubation period (time-0) and after 4 days of incubation under the conditions shown at the bottom of the figure. Islets were sonicated in PBS +0.05% BSA and kept at −20°C overnight. Islet insulin content was measured by radioimmunoassay. Data are means±SEM from n = 3 with duplicate samples in each experiment. (**B**) Human islets (100) were cultured for 4 days in cCMRL, 8 mM glucose+MSDC-0160, LiCl or IGF-1 as indicated. ^3^H-thymidine was added to each dish 24 h before the end of the 4-day period. Data are the means±SEM from n = 3, with duplicate samples in the experiment. Data analyzed using one-way ANOVA followed by Newman-Keuls Post-hoc test. (**C**) MSDC-0160 decreases Wnt signaling pathway by blocking β-catenin nuclear translocation. Immunohistochemical staining of insulin and β-catenin in human islets cultured for 4 days in CMRL containing 5 or 8 mM glucose±MSDC-0160 or LiCl as indicated. Upper panel shows the merged image of β-catenin (red), insulin (green) and DAPI (blue). The white square in each image of the upper panels is enlarged and displayed in detail in the lower panel (inset). White arrows show nuclear β-catenin. Images were acquired at 40X magnification and are representative of n = 3 experiments. Scale bar = 50 µm. Insulin = green, Pdx1 = Red, DAPI = blue. (**D**) Sequential strategy to enhance DNA synthesis and preserve insulin content in human islets. Islets were cultured in cCMRL, 8 mM glucose+LiCl for 4-day as indicated. After a 4 day stimulatory period, medium was replaced with 5 mM glucose+MSDC-0160 and IGF-1 for another 4-day period (total 8-days of incubation). Islet insulin content was measured from 20 islets in duplicates at the start of the incubation period (time-0), after 4 days and 8 days. Islets were sonicated in PBS +0.05% BSA and kept at −20°C over night. Islet insulin content was measured by RIA. Data are means±SEM from n = 3 with duplicate samples in each experiment.

Fig. 5A shows that insulin content significantly declined over a 4-day period when human islets were cultured in the presence of 8 mM glucose alone, or 8 mM glucose in the presence of IGF-1 or LiCl compared to insulin content of islets on Day 0 (column 1 vs. 3, 4 and 7). In contrast, islets cultured with 8 mM glucose and MSDC-0160± IGF-1, conditions that downregulate mTOR activity, resulted in an ∼3-fold increase in insulin content compared to 8 mM glucose+IGF-1 (column 4 vs. 5 and 6). However, these actions of MSDC-0160 were significantly blocked by the co-incubation with 5 mM LiCl that activates Wnt signaling (columns 7–9). Therefore, activation of Wnt signaling enhances human islet DNA synthesis, but blocks the preservation of insulin content by MSDC-0160.

Next, we determined the effects of Wnt signaling activation in the presence of MSDC-0160 on DNA synthesis. In Fig. 5B, LiCl significantly increased DNA synthesis compared to 8 mM glucose alone (column 1 vs. 2). In contrast, MSDC-0160± LiCl or IGF-1 did not increase DNA synthesis (columns 3–8). Overall, activation of Wnt signaling has pro-growth effects, and MSDC-0160 promotes preservation of insulin content, whereas the combination of these compounds diminishes both effects.

It is recognized that the DNA synthesis experiments in this study were conducted using intact human islets that are composed of several cells types, in addition to β-cells. DNA synthesis is an indicator and prerequisite for cell cycle progression and proliferation but in intact islets limits the interpretation of the data. In our previous study [7] we quantified β-cell proliferation by immunohistochemistry in response to LiCl that was also associated with increases in DNA synthesis and cell cycle progression. In that study, there was a significant increase in insulin and Ki-67 co-stained cells. Based on the previous data, we expect that the changes are representative of effects in β-cells.

### MSDC-0160 Inhibits β-catenin Nuclear Translocation

Previously, we observed that a decrease in mTOR with rapamycin blocks β-catenin nuclear translocation [7]. For this reason, we performed IHC analysis of β-catenin in human β-cells. Fig. 5C column 4, shows that treatment with 8 mM glucose+LiCl resulted in nuclear translocation of β-catenin in β-cells. A majority of these nuclear β-catenin positive cells show decreased staining for insulin (marked by arrows) suggestive of loss of β-cell phenotype. In contrast, treatment with MSDC-0160 prevented nuclear translocation with β-catenin localized on the cell membrane or cytoplasm (indicated by membranous staining of β-catenin, column 3) and these islets had increased insulin staining. In column 5, LiCl and MSDC-0160 together counteract each other resulting in both decreased insulin staining and decreased nuclear β-catenin translocation. Overall, Figs. 4 and 5 suggest that MSDC-0160 treatment mediates downregulation of Wnt signaling by preventing β-catenin nuclear translocation and restores insulin sensitivity. Thus, strategies to stimulate DNA synthesis and preservation of insulin content simultaneously may not be feasible, at least in vitro.

### Sequential Model to Enhance DNA Synthesis and Preservation of Insulin Content of Human Islets

As indicated in Fig. 5D, insulin content of islets was initially determined and designated time-0. Treatment of islets with 8 mM glucose and 5 mM LiCl for 4 days produced a marked decline in insulin content (4.2 ng/islet; black column) in comparison to the insulin content in islets at time-0 (18.7 ng/islet; white column) although DNA synthesis was increased under these same conditions (Fig. 5B, column 2). As shown in Fig. 5D, column 2, after the 4-day period in the pro-growth media (8 mM glucose +5 mM LiCl), the media was replaced with cCMRL that contained 5 mM glucose ± MSDC-0160± IGF-1 for 4 additional days (Days 5–8). The transfer of these islets to basal glucose (5 mM) alone or with IGF-1 (Fig. 5D, columns 3&4), partially, but significantly restored insulin content (11.3 ng/islet). However, the presence of MSDC-0160 completely restored insulin content to the level of insulin extracted from islets at time-0 (18.8 ng/islet) irrespective of the addition of IGF-1 (Fig. 5D, columns 5&6).

## Discussion

This investigation sought to reduce resistance in the insulin signaling pathway and regulate nutrient sensing pathways to achieve a balance between regenerative processes and maintenance of the β-cell phenotype in intact adult human islets in vitro. A recent study reported that β-cell dedifferentiation resulted in degranulated β-cells with decreased insulin content and this might be a mechanism for β-cell failure in T2D [34]. Other studies suggested that the removal of the negative feedback involving Ser/Thr phosphorylation of IRS-2 protects mouse islets from proinflammatory cytokines and enhances β-cell function under stress [35]. Preserving or restoring the β-cell phenotype may thus prove to be an effective strategy to treat β-cell dysfunction.

In this study, we used a new generation insulin sensitizer TZD with diminished PPARγ binding but which has proven anti-diabetic activity in phase 2 clinical studies [33]. Treatment of human islets with this compound produced a dose-dependent decrease in mTOR activation and was associated with restoration of the insulin signaling pathway. The insulin signaling pathway and Pdx1 are also known to regulate β-cell survival and expansion. Johnson et al. provided evidence for insulin action on Akt phosphorylation and Pdx1 translocation that serves as a master regulator of islet survival in human and mouse β-cells [36]. There are also similarities in the expression of *insulin* and *Pdx1* between immature embryonic β-cell progenitors and a proportion of adult β-cells [37]. In addition, metabolism of nutrients, resulting in insulin secretion in pancreatic β-cells activates Pdx1 and the insulin promoter in a PI3-K dependent manner, suggesting a role of insulin signaling in regulating Pdx1 activity [38]. Our data demonstrates that MSDC-0160 restored IGF-1-induced phosphorylation of Akt and GSK-3β, and increased both gene and protein expression of Pdx1. Importantly, MSDC-0160 and IGF-1 treatment restored insulin content and increased gene expression of other β-cell specific markers, *insulin, nkx6.1, nkx2.2*, indicating preservation of the β-cell phenotype. Our data also demonstrated that MSDC-0160 reduces glucose-induced increases in markers of apoptosis in human islets. It is probable that both prevention of apoptosis and restoration of insulin content are occurring with MSDC-0160+ IGF-1 treatment in human islets.

Our studies showed that activation of Wnt signaling with a GSK-3 inhibitor blocked the ability of MSDC-0160 to maintain insulin content, inhibited the expression of β-cell specific genes, and blocked the positive effects of MSDC-0160 on Akt phosphorylation and Pdx1 expression. Conversely, MSDC-0160 blocked Wnt signaling mediated increases in DNA synthesis. Thus, counteracting pathways are involved during the processes of DNA synthesis and preservation of the β-cell phenotype. Studies on the transcriptional regulator, HES-1 or the RNA binding protein, Musashi (MSI-1) of the notch signaling pathway, also point to other transcriptional control mechanisms that have a suppressive role in insulin gene expression while exhibiting a proliferative effect [3], [39]. In addition, cultured human islets with considerable proliferative capacity were previously reported to be insulin negative [40]–[42]. It has been suggested that switch factors may exist that regulate a shift between the adaptive responses of insulin granule biogenesis and hyperplasia [9]. In brown adipose tissue (BAT) progenitor cells, MSDC-0160 steered cells towards terminal differentiation by blocking Wnt signaling. Conversely, MSDC-0160’s effects on differentiation were lost with forced activation of the Wnt pathway (unpublished data, RFK). Therefore, the investigators hypothesized that in BAT progenitor cells, the action of MSDC-0160 is associated with the downregulation of Wnt signaling that allows cells to exit the cell cycle and undergo differentiation.

In our current study, activation of Wnt signaling by GSK-3 inhibition results in β-catenin translocation, driving DNA synthesis. In contrast, downregulation of mTOR activity by MSDC-0160 appears to set a brake in Wnt signaling by inhibiting β-catenin nuclear translocation. This was similar to our previous findings that rapamycin prevented mTOR-mediated β-catenin nuclear translocation in human β-cells [7]. Rapamycin has also been reported to inhibit β-catenin accumulation and nuclear translocation in keratinocytes and peritoneal mesothelial cells [43], [44]. Our studies using MSDC-0160 also indicate that strategies to simultaneously promote DNA synthesis and preservation of the β-cell phenotype in vitro may not be feasible. Therefore, we explored a sequential strategy to first induce DNA synthesis, a prerequisite for β-cell proliferation, followed by treatment with MSDC-0160± IGF-1, resulting in restoration of insulin content. This type of strategy could prove useful in optimizing pretreatment of human islets ex vivo for use in transplantation protocols.

**Figure 6:**
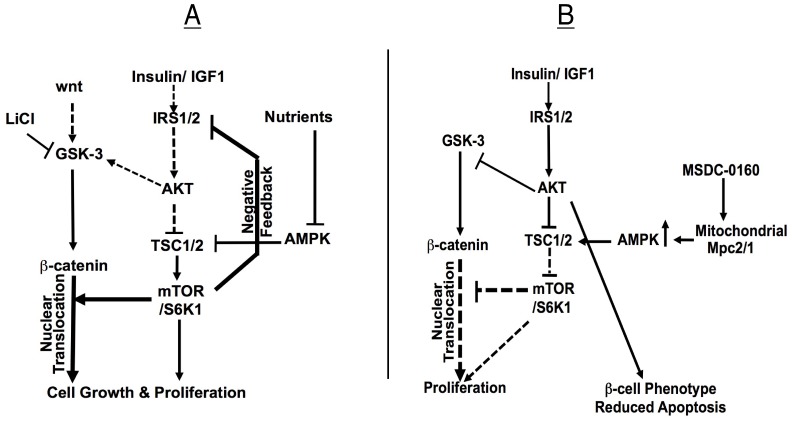
Proposed pathways involved in regenerative processes and preservation of the β-cell phenotype in human islets in vitro. mTOR is pivotal in regulating a balance between proliferation and differentiation. Human islets (left panel), display a high level of insulin signaling pathway resistance due to chronic mTOR activation. This results in negative feedback, IRS-1/2 degradation and the inability of Akt to inhibit GSK-3 and engage the Wnt/β-catenin pathway. To enhance human β-cell proliferation, pharmacologic inhibition of GSK-3, in combination with mTOR-mediated β-catenin nuclear translocation is required. To shift this balance in favor of maintaining β-cell differentiation (right panel), GSK-3 inhibitors are removed and islets are treated with the PPARγ sparing TZD, MSDC-0160 that enhances AMPK activity, downregulates mTOR/S6K1-dependent negative feedback and restores the insulin-signaling pathway. Activation of Akt by insulin/IGF-1 results in increases in differentiation markers, *insulin*, *pdx-1*, *nkx6.1* and *nkx2.2,* promotes β-cell survival and restores insulin content. Downregulation of mTOR also prevents β-catenin translocation to the nucleus, blocking DNA synthesis. Thus, DNA synthesis and differentiation do not occur simultaneously. MSDC-0160 may produce a unique modulation of nutrient sensors that give rise to signals that cause β-cells to leave the cell cycle and differentiate (right panel). The cycling of proliferation, followed by treatment with MSDC-0160 to allow restoration of insulin content, may maintain a balance between regenerative processes and preservation of the β-cell phenotype. Dotted line indicates reduced signaling levels.

Our proposed model for regulating a balance between growth, survival and the maintenance of the β-cell phenotype is represented schematically in Fig. 6. Due in part to chronic mTOR activation, isolated human islets rarely engage the Wnt/β-catenin or insulin signaling pathways. As a result, neither regenerative processes nor β-cell specific gene expression are engaged in vitro (Fig. 6A). In this proposed model resistance in the insulin signaling pathway is circumvented by GSK-3 inhibition that increases Wnt signaling, β-catenin nuclear translocation, DNA synthesis and β-cell proliferation. However, resistance in the insulin signaling pathway, due to chronic mTOR activation, is still present, resulting in a loss of insulin content and decreased survival.

Fig. 6B describes our approach to restore insulin signaling pathway sensitivity. In our model, the GSK-3 inhibitor that promotes proliferation is removed and MSDC-0160+ insulin or IGF-1 are added. MSDC-0160 downregulates mTOR, in part through AMPK activation but other targets may be involved. Our data show that MSDC-0160 produces a dose-dependent activation of AMPK that correlates with decreases in mTOR activity. The downregulation of mTOR with MSDC-0160 blocks β-catenin nuclear translocation that prevents DNA synthesis and proliferation. In addition, the reduced mTOR activity removes the negative feedback to the insulin signaling pathway and allows insulin/IGF-1 induced Akt phosphorylation. This results in increased β-cell specific gene expression and increased survival.

The anti-diabetic class of TZD drugs and metformin, activators of AMPK, are suggested to regulate mTOR and the insulin signaling pathway in various cells in part through inhibition of mitochondrial respiratory complex 1 [22], [45], [46]. However, TZDs and metformin have different pharmacologies and may have other ways of activating AMPK [47], [48]. In clinical studies of type 2 diabetes, patients treated with metformin [49] or in isolated adipocytes from patients treated with TZDs [50], there were distinct effects of both drugs on the PI-3 K-dependent pathway. TZDs increased the activity of PI-3K dependent pathways thereby increasing the number of glucose transporters at the cell surface in contrast to metformin. Metformin also had predominant effects on inhibition of hepatic glucose production [49]. Therefore TZDs may be preferable to metformin for preservation of β-cell mass.

MSDC-0160 is a prototype member of a new class of insulin sensitizers designed to maintain binding to a mitochondrial target (mTOT) and has been shown to lower glucose as effectively as pioglitazone in a phase 2 clinical trial [33]. mTOT is a previously unrecognized protein complex containing two key proteins, BRP44 and BRP44-like [51]. These proteins have been recently renamed, Mpc2 and 1 respectively [52]–[54], because they appear to function at least in part, as a mitochondrial pyruvate carrier. Recent results have identified Mpc1 and Mpc2 in Drosophila and mammalian tissues as components of the mitochondrial target of TZDs (mTOT) [55]. We have conducted studies in human islets to determine if these proteins are also expressed in human islets. Competitive binding of MSDC-0160 with a ^125^I-labeled photoaffinity crosslinking probe to Mpc2 suggests the presence of this complex in human islets (Fig. S4A). In addition, immunoblot and immunochemical analysis suggests that the Mpc2 protein resides in the mitochondria and is expressed in human β-cells (Fig. S4 B&C). Future studies will further examine the mitochondrial metabolic signals generated from MSDC-0160 modulation of the mTOT complex in human β-cells and to determine whether or not the differentiation or anti-apoptotic mechanisms described here are through modification of this target.

Overall, our study has provided evidence that regulation of mTOR by a novel insulin sensitizer, MSDC-0160, restores sensitivity in the insulin signaling pathway, promotes human β-cell differentiation and reduces the expression of markers of apoptosis. Our data also highlights the inability of isolated human islets to simultaneously promote DNA synthesis and differentiation. Hence, we demonstrate that an ex-vivo sequential strategy of alternating induction of DNA synthesis, followed by treatment with MSDC-0160 to promote differentiation and reduce markers of apoptosis can preserve insulin content and β-cell phenotype.

## Supporting Information

Figure S1 **Effects of MSDC-0160 on** ***insulin*** **and** ***pdx-1*** **gene expression in human islets.** Human islets were cultured for 24 h in cCMRL, 5 or 8 mM glucose±MSDC-0160, insulin and LiCl as indicated. The islets were harvested for RNA isolation, cDNA synthesis and real-time PCR analysis. Taqman probes with primers specific for *insulin* and *pdx-1* genes were used. Target genes were quantified by relative quantification using the comparative Ct method. Data are means ± SEM of n = 3–4 experiments. Data was analyzed using one-way ANOVA followed by Newman-Keuls test.
(TIF)

Figure S2 **MSDC-0160± IGF-1 does not increase ERK phosphorylation.** Western Blots for phosphorylated, and total ERK in human islets cultured for 24 h in CMRL containing 5 mM glucose±MSDC-0160± IGF1 as indicated. Top panel shows representative Western blot and bottom panel shows mean values after quantitation of the densitometry. Data are means ± SEM from n = 2 experiments.
(TIF)

Figure S3 **The effects of MSDC-0160 and IGF-1 on glucose-induced apoptotic gene and protein expression markers in human islets.** Islets were cultured for 4-days in cCMRL, glucose (5, 8, 16.5, or 33.3 mM) with or without the concentrations of MSDC-0160 and IGF-1 as indicated. The islets were harvested for Western blot or RNA isolation followed by cDNA synthesis and real-time PCR analysis. **(A,C–F)** Taqman probes with primers specific for *bcl-2, bax* and *caspase-3* were used. Target genes were quantified by relative quantification using the comparative Ct method. Black squares = *bcl-2*, black circles = *caspase-3*, black triangles = *bax*. **(B,G)** Western blots for cleaved caspase-3 were quantitated by densitometry and normalized against β-actin. Data are means ± SEM of n = 3 experiments. Data was analyzed using one-way ANOVA followed by Newman-Keuls test.
(TIF)

Figure S4 **Identification of mitochondrial target of MSDC-0160 in human and rat islets. (A)** Binding of MSDC-0160 to its mitochondrial target protein. Human islets (∼1000–1500/sample) were cultured for 4 days in cCMRL, 8 mM glucose, crude mitochondrial pellets were prepared and 10 µg of protein (mitochondrial P2 fraction) was incubated with a crosslinking probe that labels Mpc2 with or without the concomitant addition of 20 µM MSDC-0160. The arrows show the position of Mpc2 and the reduction of crosslinking on addition of MSDC-0160. Data is representative of n = 2 with triplicate samples in each experiment. **(B)** Presence of Mpc2 protein in the mitochondrial fraction of rat islets and liver tissue as indicated. Mitochondrial extracts (P2) of both islet and liver tissues from rats were prepared as described in Methods. P2 fractions were processed for Western blotting. Complex III was used as a loading control and an indicator of mitochondrial fraction. Data representative of n = 2 experiments **(C)** Cellular localization of Mpc2 and Mpc1 in the mitochondria of human β-cells. Human islets were cultured with cCMRL containing 8 mM glucose for 4 days. Islets were then dispersed and placed on slides using a Cytospin Centrifuge. Left panel indicates the presence of Mpc2 (upper) and Mpc1 (lower) protein (green) in the cytoplasm co-localizing with Mitotracker (red) in the islet cells. Right panel demonstrates the presence of Mpc2/Mpc1 (red) in the cytoplasm of human β-cells stained for insulin (green). Red staining (mitochondrial) distinct from green (insulin granules) in the β-cells can be noted. A blue nucleus represents DAPI staining. Images were taken at 100× magnification. Scale bar = 10 µm. Images are representative of n = 3 independent experiments.
(TIF)
