# Supplementary figures and images for: Novel Insulin Sensitizer Modulates Nutrient Sensing Pathways and Maintains β-Cell Phenotype in Human Islets
Source: PLoS One. 2013 May 1;8(5):e62012. doi: 10.1371/journal.pone.0062012 (PMC3641131; doi:10.1371/journal.pone.0062012)

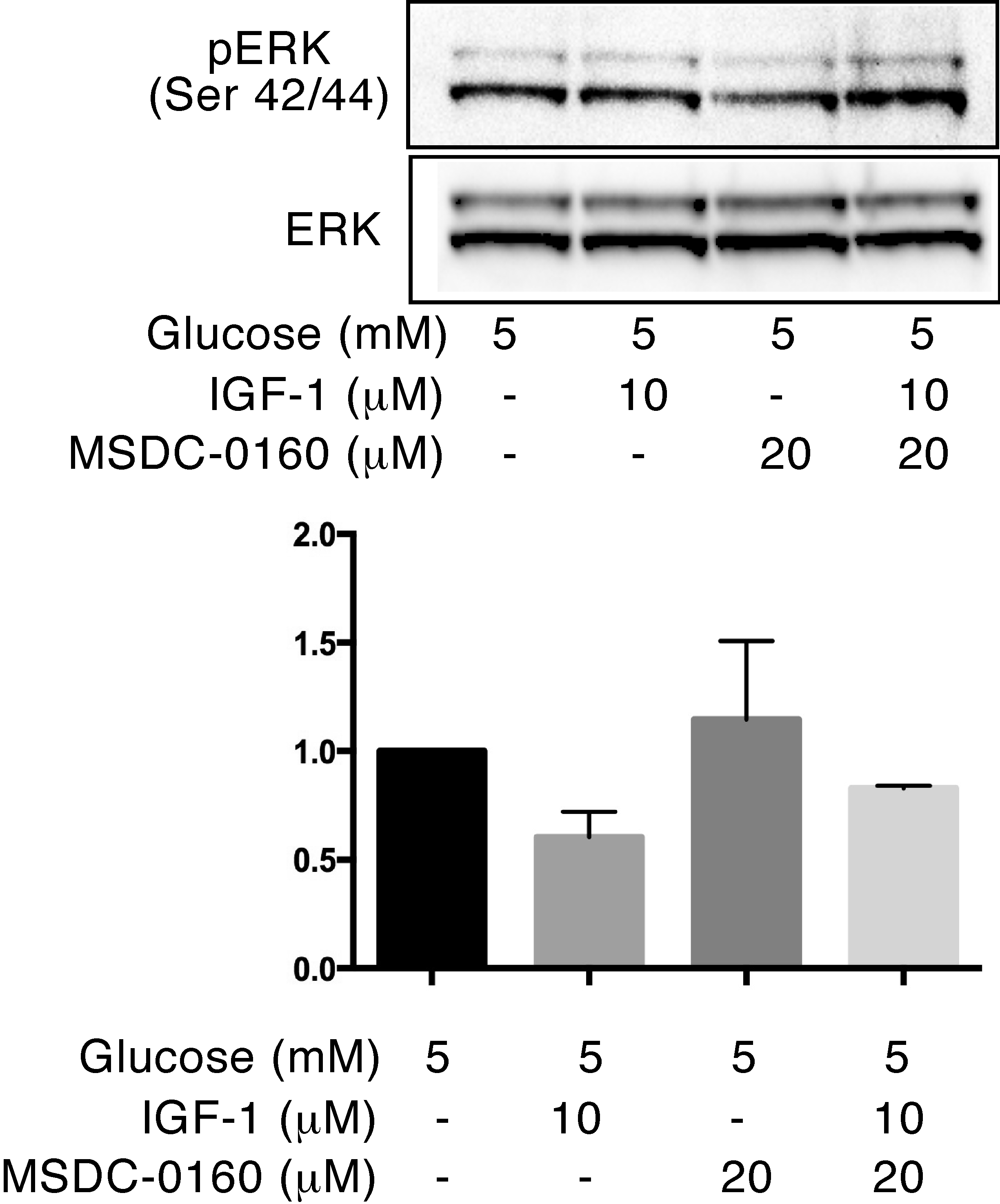

Supplement: Figure S2 — MSDC-0160± IGF-1 does not increase ERK phosphorylation. Western Blots for phosphorylated, and total ERK in human islets cultured for 24 h in CMRL containing 5 mM glucose±MSDC-0160± IGF1 as indicated. Top panel shows representative Western blot and bottom panel shows mean values after quantitation of the densitometry. Data are means ± SEM from n = 2 experiments. (TIF) [file pone.0062012.s002.tif]

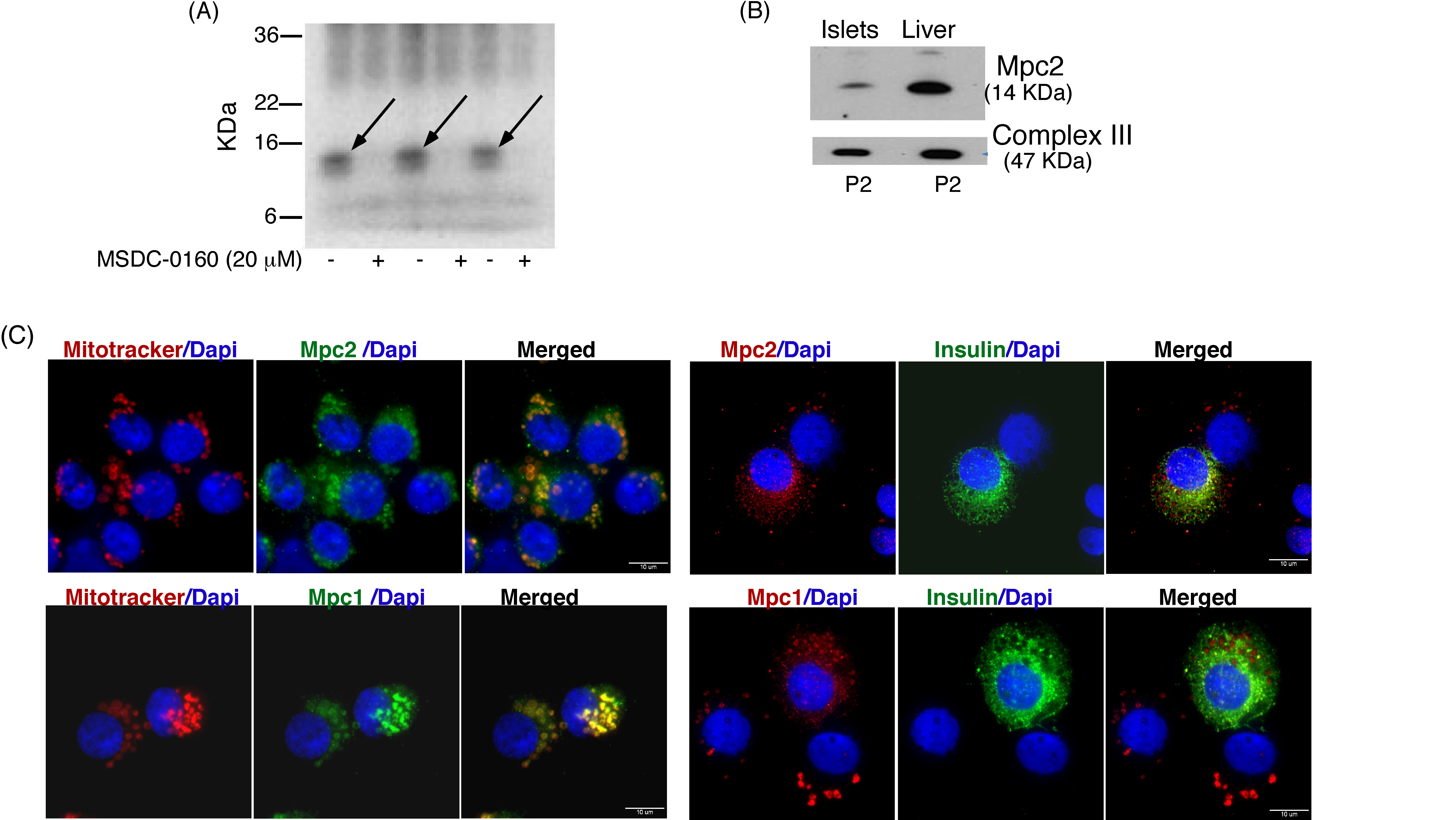

Supplement: Figure S4 — Identification of mitochondrial target of MSDC-0160 in human and rat islets. (A) Binding of MSDC-0160 to its mitochondrial target protein. Human islets (∼1000–1500/sample) were cultured for 4 days in cCMRL, 8 mM glucose, crude mitochondrial pellets were prepared and 10 µg of protein (mitochondrial P2 fraction) was incubated with a crosslinking probe that labels Mpc2 with or without the concomitant addition of 20 µM MSDC-0160. The arrows show the position of Mpc2 and the reduction of crosslinking on addition of MSDC-0160. Data is representative of n = 2 with triplicate samples in each experiment. (B) Presence of Mpc2 protein in the mitochondrial fraction of rat islets and liver tissue as indicated. Mitochondrial extracts (P2) of both islet and liver tissues from rats were prepared as described in Methods. P2 fractions were processed for Western blotting. Complex III was used as a loading control and an indicator of mitochondrial fraction. Data representative of n = 2 experiments (C) Cellular localization of Mpc2 and Mpc1 in the mitochondria of human β-cells. Human islets were cultured with cCMRL containing 8 mM glucose for 4 days. Islets were then dispersed and placed on slides using a Cytospin Centrifuge. Left panel indicates the presence of Mpc2 (upper) and Mpc1 (lower) protein (green) in the cytoplasm co-localizing with Mitotracker (red) in the islet cells. Right panel demonstrates the presence of Mpc2/Mpc1 (red) in the cytoplasm of human β-cells stained for insulin (green). Red staining (mitochondrial) distinct from green (insulin granules) in the β-cells can be noted. A blue nucleus represents DAPI staining. Images were taken at 100× magnification. Scale bar = 10 µm. Images are representative of n = 3 independent experiments. (TIF) [file pone.0062012.s004.tif]
